# Blood eosinophil reference values and determinants in a representative adult population

**DOI:** 10.1016/j.jacig.2025.100449

**Published:** 2025-03-08

**Authors:** Reshed Abohalaka, Selin Ercan, Lauri Lehtimäki, Saliha Selin Özuygur Ermis, Daniil Lisik, Muwada Bashir Awad Bashir, Radhika Jadhav, Linda Ekerljung, Göran Wennergren, Jan Lötvall, Teet Pullerits, Helena Backman, Madeleine Rådinger, Bright I. Nwaru, Hannu Kankaanranta

**Affiliations:** a Krefting Research Centre, Department of Internal Medicine and Clinical Nutrition, Institute of Medicine, Sahlgrenska Academy, University of Gothenburg, Gothenburg, Sweden; b Allergy Centre, Tampere University Hospital, Tampere, Finland; c Faculty of Medicine and Health Technology, Tampere University, Tampere, Finland; d Department of Internal Medicine/Respiratory Medicine and Allergology, University of Gothenburg, Gothenburg, Sweden; e Department of Paediatrics, Sahlgrenska Academy, University of Gothenburg, Gothenburg, Sweden; f Department of Public Health and Clinical Medicine, Umeå University, Umeå, Sweden; g Department of Respiratory Medicine, Seinäjoki Central Hospital, Seinäjoki, Finland

**Keywords:** Blood eosinophil count, asthma, COPD, atopy, allergy, reference values, population-representative, normal range

## Abstract

**Background:**

The use of blood eosinophil count (BEC) as a prognostic biomarker in the management of conditions such as asthma and chronic obstructive pulmonary disease (COPD) may be complicated by factors such as atopy, age, sex, smoking, and comorbidities.

**Objective:**

We sought to produce reference values for BEC, considering age, asthma, COPD, and clinical allergy for the general adult population.

**Methods:**

The West Sweden Asthma Study constitutes a population-representative clinical epidemiological cohort of randomly selected adults in Western Sweden. From this cohort, 1145 individuals took part in clinical examinations, including skin prick testing, specific IgE, and BEC.

**Results:**

The upper limit (95th percentile) of BEC varied by age. It ranged from 400 to 500 cells/μL in the full sample and from 300 to 400 cells/μL in subjects without asthma, COPD, and clinical allergy (n = 710). Sex, smoking, atopy, clinical allergy, obesity, asthma, COPD, diabetes, and hypertension were statistically significantly associated with higher BEC levels. However, only asthma and clinical allergy in the full sample, and obesity and diabetes in those without asthma, COPD, or clinical allergy, remained statistically significant with higher BEC levels in multivariable regression analyses.

**Conclusions:**

In a population-representative sample, the upper limit of BEC in healthy adults ranged between 300 and 400 cells/μL, varying by age. Age, smoking, obesity, asthma, COPD, and clinical allergy influence BEC levels and should be considered in clinical interpretation.

Blood eosinophil count (BEC) plays a pivotal role in determining the severity, phenotypes, and therapy of a spectrum of chronic inflammatory lung disorders, most notably asthma and chronic obstructive pulmonary disease (COPD).^1^^,^^2^ Indeed, BEC is a valuable prognostic indicator for assessing responsiveness to anti–IL-5 and anti–IL-4/IL-13 therapies in severe asthma^3^ as well as for predicting the efficacy of inhaled corticosteroids (ICSs) in COPD.^4^^,^^5^ In addition, the Global INitiative for Asthma recommends the use of BEC to identify asthmatic patients with type 2 inflammation.^2^ Meanwhile, the Global Strategy for the Diagnosis, Management and Prevention of Chronic Obstructive Lung Disease recommends using a threshold of 300 cells/μL of blood eosinophils for patients with COPD, or 100 cells/μL for those who continue to experience exacerbations despite appropriate bronchodilator therapy, to guide the use of ICSs in COPD treatment.^1^

However, the application of BEC as a biomarker in clinical practice is not straightforward. First, the predictive cutoff points for BEC interpretation derived from randomized controlled trials are confined to studies conducted within asthma and COPD populations.^6^, ^7^, ^8^, ^9^, ^10^, ^11^, ^12^, ^13^, ^14^, ^15^, ^16^ Second, the recommended BEC thresholds in clinical guidelines are subject to debate because of their perceived overlap with normal eosinophil ranges.^17^^,^^18^ This controversy arises from the limited evidence of normal and abnormal blood eosinophil levels across diverse populations with varying backgrounds and health conditions. This evidence gap is highlighted by the fact that investigations into healthy populations suggest that BEC is influenced by several demographic and clinical factors, including age, sex, obesity, atopy, and smoking.^19^, ^20^, ^21^

Furthermore, studies having representative random samples from the general population are scarce. A recent systematic review by Benson et al^21^ that aimed to synthesize the absolute BEC, rather than reference values, identified 14 studies conducted in the general population. Of these studies, only 1 study used a random sample directly from the population itself.^22^ Conversely, 2 additional studies, although encompassing a considerable sample size, did not recruit participants from the general population^20^ nor did report reference values for the healthy cohort.^23^ Thus, the typical range of BEC in the general population is not well established. Therefore, our objective was to determine the BEC reference values and their determinants in a population-representative sample. Parts of this work have been published as an abstract.^24^^,^^25^

## Methods

### Study area and population

**Fig 1:**
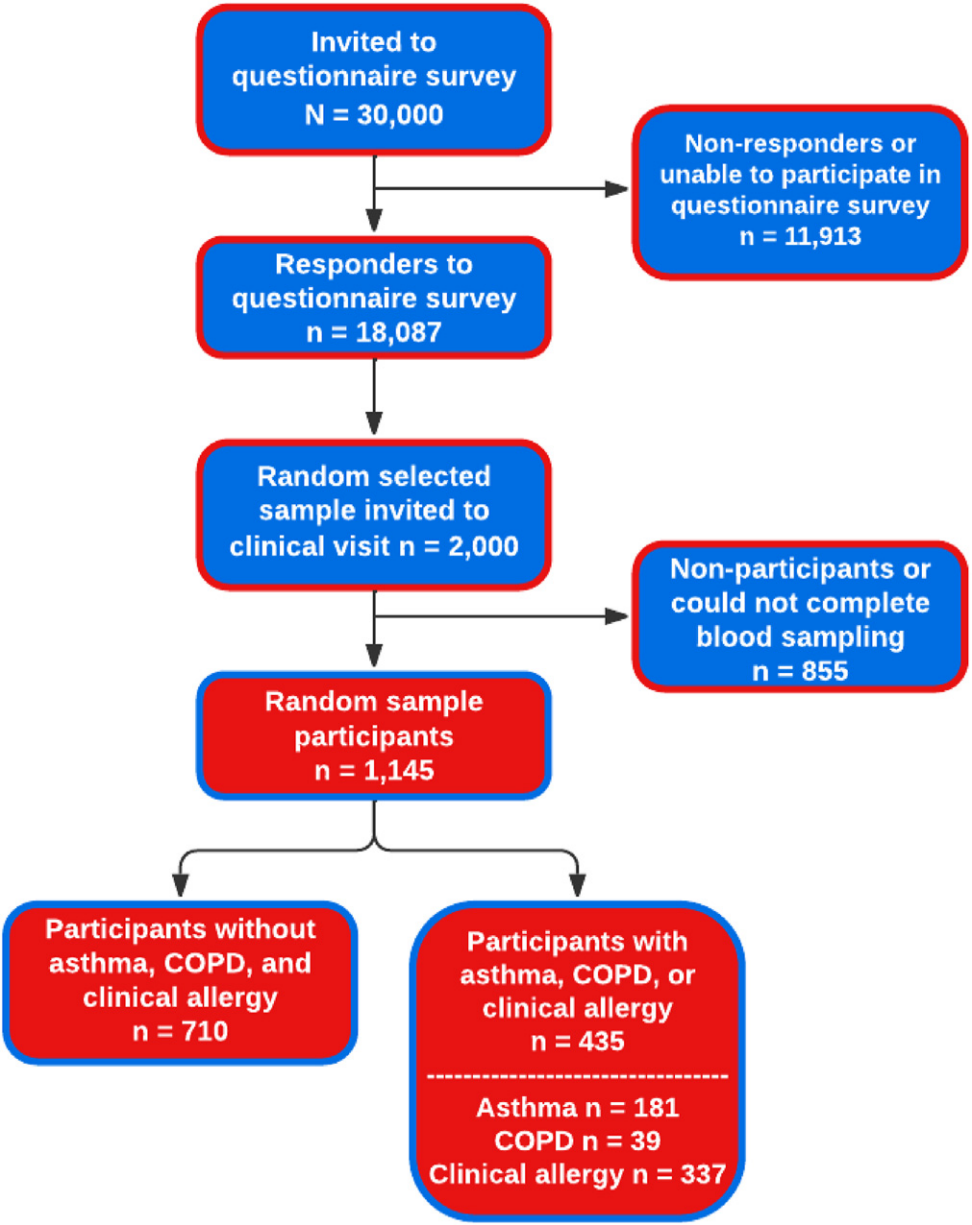
Flow chart of participation in the study. Clinical allergy was defined by the presence of allergic sensitization coupled with consistent self-reported allergic symptoms attributable to the same allergen.

The West Sweden Asthma Study (WSAS) has been described in detail previously.^26^ Briefly, the WSAS examines individuals aged 16 to 75 years at recruitment, randomly selected from the general population of Western Sweden. Commencing in 2008, a total of 30,000 subjects within the specified age range were randomly selected through the Swedish Population Register and invited to take part in a postal survey. Of those invited, 18,087 individuals participated in the survey study. Subsequently, a random subset of 2,000 individuals was invited to comprehensive clinical examinations, of whom 1,145 participated (Fig 1). All participants provided informed consent, and the study was approved by the regional ethics board in Gothenburg, Sweden.

### Clinical examinations

The examinations included, but were not limited to, blood cell quantification, skin prick testing (SPT), specific IgE (sIgE) level assessment, and measurement of height and weight. In addition, the clinical examination encompassed in-depth, structured interviews and administration of questionnaires pertaining to respiratory diseases and symptoms, morbidities, health care utilization, and potential risk factors.

### Assessment of sensitization and blood eosinophils

Sensitization was evaluated through the determination of sIgE levels and SPT for 11 aeroallergens (details of which are provided in this article’s Online Repository at www.jaci-global.org). BECs were determined using standard procedures at Sahlgrenska University Hospital (Gothenburg, Sweden) with the ADVIA 2120i Hematology System (Siemens Healthineers, Erlangen, Germany) and are reported as the number of cells per microliter.

### Definitions of diseases

Clinical allergy was defined as the presence of allergic sensitization, indicated by either a positive SPT result or an elevated sIgE level to at least 1 allergen (atopy), coupled with self-reported allergic symptoms attributable to the same allergen. The presence of metabolic disorder was confirmed by obesity (body mass index [BMI], ≥30 kg/m^2^) or any of the following self-reported conditions: hypertension, hyperlipidemia, and diabetes.^27^ Detailed definitions of asthma and COPD are provided in the Online Repository. Individuals who were free from asthma, COPD, and clinical allergies were considered as “healthy.”

### Statistical analyses

In the establishment of reference values, the presumption is that the spectrum of values derived from a cohort of healthy individuals is a proxy for normal values. Consequently, individuals manifesting values beyond the norm, typically delineated by the central 90% of the range of values in the healthy population (upper limit of normal or 95th percentile and lower limit of normal or 5th percentile), are frequently characterized as exhibiting anomalous outcomes.^28^^,^^29^ As a result, our reporting considers the 95th percentile of BEC as the upper limit of normal. Because of the nonnormal distribution of BEC values exhibiting a right-skewed shape, percentiles were derived from the logarithmically transformed data set, as previously described.^20^ Statistical comparisons were carried out using independent Student *t* tests. A *P* value less than .05 was considered statistically significant.

A multivariable binary logistic regression model was applied to compute odds ratios accompanied by 95%CI for the purpose of elucidating associations between various factors and BEC values higher than the upper limit (95th percentile), adjusting for potential confounding variables. The normal weight range (20 ≤ BMI < 25) served as the reference for BMI-related variables, and the age group of 30 to 60 years was used as the reference category for age variables, given that allergic conditions and elevated BEC levels are more common in younger (<30 years) and older (>60 years) individuals.^20^^,^^27^ All statistical analyses were executed using SPSS 29.0 (IBM Corp, New York, NY).

## Results

### Characteristics of the random sample

The random sample comprised 1145 individuals, with 46.8% being male. The mean age of the total study sample was 50.4 ± 15.4 years. Nearly half the participants (50.8%; n = 582) reported no history of smoking, whereas 11.4% were current smokers. Of the participants, 37.8% (n = 426) were sensitized (atopic), whereas 29.8% (n = 337) met the criteria for clinical allergy (Table I). Individuals with clinical allergy were younger (45.6 ± 14.7 years) than those without clinical allergy, and more of them were male and nonsmokers (Table I). For other characteristics of those with asthma with or without clinical allergy, see Table E1 (in the Online Repository available at www.jaci-global.org).

**Table I:** Characteristics of study subjects with or without asthma or clinical allergy (N = 1145)

Characteristics	Without asthma	With asthma	Without clinical allergy	With clinical allergy
No. of subjects	964 (84.2)	181 (15.8)	792 (70.2)	337 (29.8)
Asthma	—	181 (100)	65 (8.2)	115 (34.1)
Clinical allergy∗	222 (23.4)	115 (63.9)	—	337 (100)
Demographic characteristics				
Age (y), mean ± SD	50.7 ± 15.5	48.7 ± 15.1	52.1 ± 15.3	45.6 ± 14.7
Sex: male	456 (47.3)	80 (44.2)	349 (44.1)	179 (53.1)
BMI (kg/m^2^), mean ± SD	26.0 ± 4.1	26.9 ± 4.5	26.1 ± 4.1	26.2 ± 4.3
Education years over 12 y	437 (45.4)	90 (50.0)	345 (43.6)	175 (52.9)
Never smokers	496 (51.5)	86 (47.5)	380 (48.0)	196 (58.2)
Current smokers	103 (10.7)	27 (14.9)	99 (12.5)	29 (8.6)
Pack year, mean ± SD	14.0 ± 12.8	17.5 ± 15.9	14.8 ± 13.1	13.3 ± 14.0
Allergic symptoms				
Age (y) at onset of symptoms, mean ± SD	24.5 ± 16.8	17.8 ± 15.0	28.6 ± 18.1	18.6 ± 14.3
Eye and nasal symptoms	249 (49.2)	106 (68.8)	119 (33.1)	234 (79.9)
Ever allergic nasal symptoms	301 (31.3)	129 (71.3)	152 (19.3)	273 (81.0)
Current allergic nasal symptoms	454 (88.0)	147 (94.8)	309 (84.5)	285 (96.0)
Atopy and allergy				
≥1 positive reaction in SPT†	223 (34.0)	102 (72.9)	60 (11.7)	265 (94.0)
≥1 positive sIgE	251 (27.4)	111 (63.4)	65 (8.5)	297 (91.7)
Atopic (SPT or sIgE)	304 (32.1)	122 (67.8)	92 (11.6)	337 (100)
Clinical allergy to mites	42 (4.4)	42 (23.3)	—	84 (24.9)
Clinical allergy to furry animals	74 (7.8)	71 (39.4)	—	145 (43.0)
Clinical allergy to pollen	168 (17.7)	97 (53.9)	—	265 (78.6)
Clinical allergy to nuts	40 (4.4)	49 (28.0)	—	89 (27.5)
Clinical allergy to seafood or dairy	38 (4.1)	19 (10.9)	—	57 (17.6)
Morbidities				
COPD	22 (2.3)	17 (9.4)	26 (3.3)	10 (3.0)
Obesity (BMI ≥ 30 kg/m^2^)	132 (13.7)	40 (22.1)	113 (14.3)	55 (16.3)
Diabetes mellitus	37 (3.8)	9 (5.0)	27 (3.4)	18 (5.4)
Hypertension	225 (23.5)	48 (26.7)	197 (25.0)	67 (20.1)
Other cardiovascular diseases	113 (11.7)	23 (12.7)	98 (12.4)	32 (9.5)
Hyperlipidemia	151 (15.7)	32 (17.7)	129 (16.3)	47 (13.9)
Metabolic disease	377 (39.1)	83 (45.9)	321 (40.5)	127 (37.7)
Gastroesophageal reflux disease	409 (42.5)	102 (56.4)	342 (43.3)	163 (48.4)

Data are presented as n (%), unless otherwise indicated.

∗ Some participants (n = 16) did not undergo SPT nor sIgE, and so they have been excluded from the total count of clinical allergy.

† Some participants (n = 349) did not undergo SPT, and so they have been excluded from the total percentage.

### BEC in the random sample

**Fig 2:**
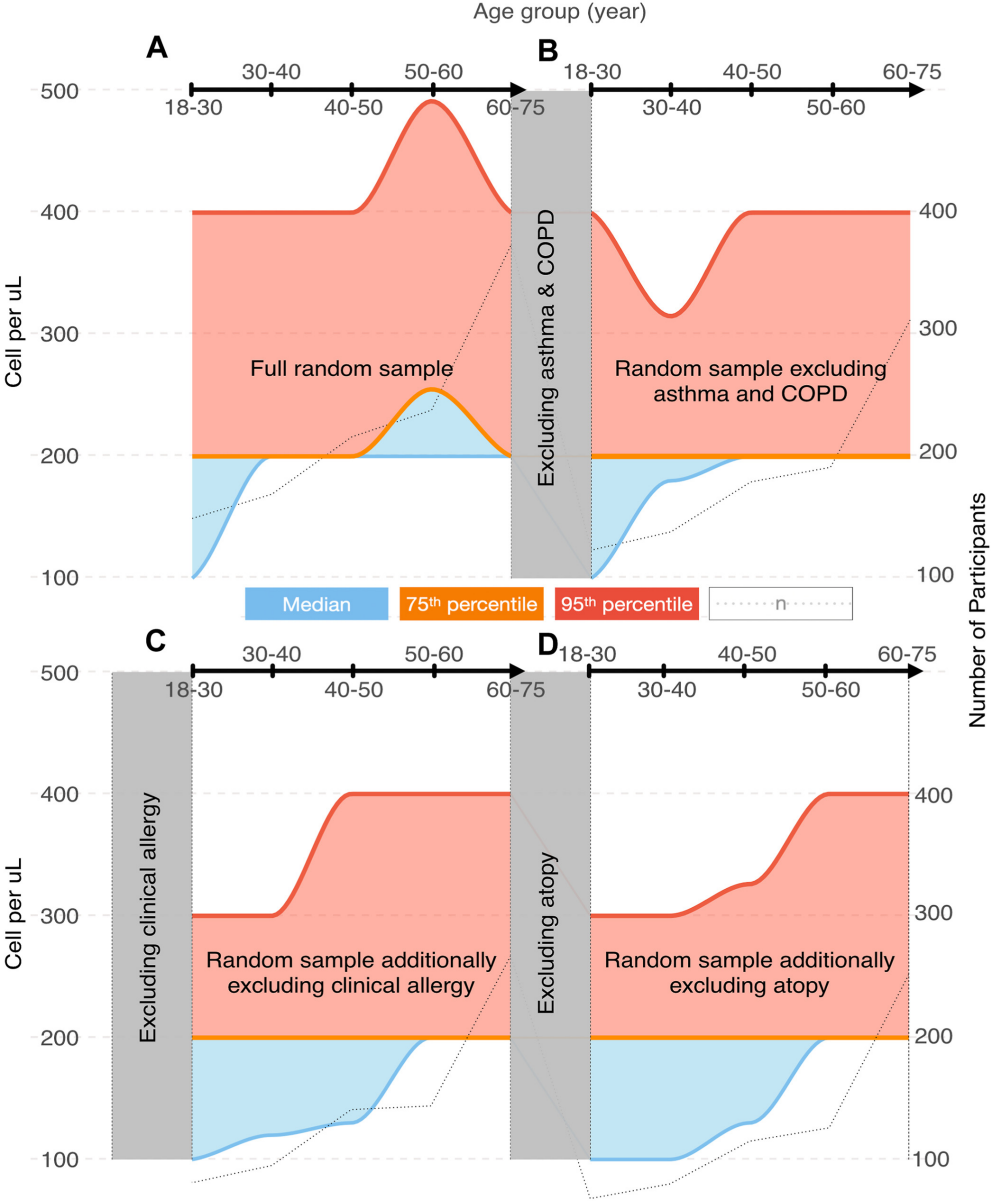
**A-D,** The characteristics of BEC across different age groups, delineated for the entire population-representative random sample of 1145 individuals (Fig 2, *A*), random sample participants excluding patients with asthma and COPD (n = 948; Fig 2, *B*), individuals additionally devoid of clinical allergy (n = 710; Fig 2, *D*), and individuals additionally devoid of atopy (n = 628; Fig 2, *D*). The depicted *blue* region demarcates the interval between the median and the 75th percentile curves, whereas the *red* region signifies the interval between the 75th and the 95th percentile curves.

In the full random sample, the upper limit of BEC was 400 cells/μL across all ages, except for those aged 50 to 60 years, for whom it reached nearly 500 cells/μL (Fig 2, *A*). To assess the influence of asthma and COPD on BEC, we excluded patients with these conditions (Fig 2, *B*). The 95th percentile BEC remained stable at 400 cells/μL for those without asthma or COPD across different ages, except for those aged 30 to 40 years, for whom it was about 300 cells/μL. Next, we examined the impact of clinical allergy on BEC (Fig 2, *C*). Among participants without clinical allergy, asthma, or COPD (n = 710), the upper BEC limit increased with age from 300 cells/μL for those younger than 40 years to 400 cells/μL for those older than 40 years (Table II). Furthermore, excluding atopic subjects, there was a minimal change in the 95th percentile compared with those without clinical allergy (Fig 2, *D*). Detailed reference values of these subjects (n = 628) are provided in Table E2 (in the Online Repository available at www.jaci-global.org).

**Table II:** The descriptive values of BEC in nonallergic, nonasthmatic, and COPD-free randomly selected participants (n = 710)

Age (y)	n	Mean ± SD (cells/μL)	P5 (cells/μL)	P25 (cells/μL)	P50 (cells/μL)	P75 (cells/μL)	P95 (cells/μL)
18-30	81	153 ± 95	50	90	100	200	300
30-40	95	154 ± 107	28	80	120	200	300
40-50	139	169 ± 112	40	100	130	200	400
50-60	140	178 ± 100	61	100	200	200	400
>60	255	175 ± 109	60	100	200	200	400

The 75th percentile of BEC was similar to the 95th percentile in the entire random sample (Fig 2, *A*). However, when excluding those with asthma, COPD, clinical allergy, or atopy, the 75th percentile remained constant at 200 cells/μL across all age groups (Fig 2, *B-D*). Meanwhile, the median BEC in the full random sample increased gradually with age, from 100 cells/μL to a peak of 200 cells/μL for those older than 30 years, with similar trends in the other groups (Fig 2).

### The impact of asthma, COPD, and clinical allergy on BEC

To understand the rise in the upper limit of normal and the 75th percentile of BEC in people aged 50 to 60 years in the random sample, we focused on the 95th and 75th percentiles of BEC in subjects with asthma, COPD, or clinical allergy. Detailed analyses of eosinophil and BEC values for the subgroups with asthma and clinical allergy in the random sample are shown in Fig E1, *A-C* (in the Online Repository available at www.jaci-global.org).

### Determinants of BEC

A comprehensive understanding of the determinants affecting the upper normal limit of BEC within the general population necessitates a comparative investigation of BEC upper 95th percentile values across diverse demographic and clinical parameters. However, the 95th percentile is a collective outcome and not individually available for each patient. Hence, to comprehend the determinants influencing BEC in the general population, we evaluated the effect of various factors, previously reported to affect BEC, on mean BEC levels in our full random sample (Table III). BEC levels were significantly higher in men than in women and in ever smokers than in never smokers. BEC levels were significantly higher in those with clinical allergy than in those without, in those with atopy than in those without, and in those with asthma than in those without. Moreover, high BEC was significantly linked to the presence of COPD, obesity, hypertension, and a minimum of 1 metabolic disease (Table III).

**Table III:** The BECs in the full random sample according to the demographic features and absence or presence of different conditions (N = 1145)

Condition	Eosinophils		*P* value
	Condition absent	Condition present	
Demographic characteristics			
Sex: male	181 ± 122	195 ± 124	**.026**
Ever smoker	178 ± 115	196 ± 130	**.007**
Current smoker	185 ± 122	205 ± 126	**.040**
Allergic symptoms			
Eye and nasal symptoms	189 ± 128	204 ± 132	.072
Ever allergic nasal symptoms	175 ± 115	206 ± 133	**<.001**
Current allergic nasal symptoms	174 ± 111	200 ± 132	.056
Current usage of antihistamines	195 ± 155	216 ± 138	.130
Atopy and allergy			
≥1 positive reaction in SPT∗	170 ± 111	210 ± 133	**<.001**
≥1 positive sIgE	176 ± 115	213 ± 136	**<.001**
Atopic (SPT or sIgE)	174 ± 113	210 ± 134	**<.001**
Clinically allergic to mites	185 ± 120	217 ± 149	**.011**
Clinically allergic to furry animals	182 ± 118	222 ± 148	**<.001**
Clinically allergic to pollen	179 ± 119	214 ± 130	**<.001**
Clinically allergic to nuts	185 ± 121	225 ± 145	**.001**
Clinically allergic to seafood or dairy	185 ± 121	244 ± 157	**<.001**
Having at least 1 clinical allergy	174 ± 113	218 ± 137	**<.001**
Morbidities			
Obesity	184 ± 123	208 ± 123	**.009**
Asthma	179 ± 115	232 ± 151	**<.001**
COPD	185 ± 120	238 ± 186	**.004**
Diabetes mellitus	186 ± 122	220 ± 148	**.031**
Hypertension	184 ± 122	198 ± 126	**.044**
Other cardiovascular diseases	185 ± 121	202 ± 135	.063
Hyperlipidemia	186 ± 121	196 ± 132	.143
Metabolic disease	182 ± 120	196 ± 127	**.030**
Gastroesophageal reflux disease	187 ± 118	187 ± 127	.487

Data are presented as mean ± SD. Statistical significances were evaluated by independent samples *t* test. Boldfaced entries signify *P* < .05 which was considered significant.

∗ Some participants (n = 349) did not undergo SPT, and so they have been excluded from this analysis.

### Multivariable linear regression model for factors associated with higher BEC values

Multivariable linear regression analyses were conducted to discern factors associated with higher BEC values considering potential confounders. To achieve this objective, 2 distinct analyses were performed: one including the entire random sample and the other focusing solely on participants lacking any predisposing factors to eosinophilic inflammation, such as allergies, atopy, asthma, or COPD. The initial analysis revealed that asthma and clinical allergy—not atopy—were significant determinants of BEC in the whole sample. This held true when adjusting for age and BMI as categorical variables (Table IV) and as continuous variables (see Table E3 in this article’s Online Repository at www.jaci-global.org).

**Table IV:** Determinants of BEC values in adjusted multivariable linear regression analysis of population-representative full random sample (N *=* 1145) and in participants free from asthma, COPD, clinical allergy, and atopy (n = 628)

Characteristics	Coefficient *B*	SE	Lower 95% CI	Upper 95% CI	*P* value
*Full random sample*					
Demographic characteristics					
Age (y)					
>60	3.56	8.95	−14.01	21.13	.691
<30	−15.31	11.41	−37.70	7.08	.180
Sex: male	8.40	7.45	−6.21	23.00	.260
BMI (kg/m^2^)					
Underweight (BMI < 18.5)	−57.22	38.62	−132.99	18.5	.139
Overweight (25 ≤BMI < 30)	3.93	8.28	−12.32	20.17	.635
Obese (BMI ≥ 30)	15.14	11.35	−7.13	37.41	.183
Ever smoking	11.94	8.06	−3.871	27.752	.139
Currently smoking	13.859	12.390	−10.45	38.17	.264
Morbidities					
Asthma	34.15	10.56	13.43	54.86	**.001**
COPD	24.74	21.25	−16.95	66.43	.245
Hypertension	5.38	9.40	−13.06	23.83	.567
Diabetes mellitus	21.24	19.22	−16.47	58.95	.269
Hyperlipidemia	−0.91	10.94	−22.37	20.56	.934
Atopy	7.42	13.31	−18.70	33.54	.577
Clinical allergy	29.88	14.25	1.93	57.84	**.036**
*Participants free from asthma, COPD, clinical allergy, and atopy*					
Demographic characteristics					
Age (y)					
>60	−0.15	9.72	−19.24	18.96	.988
<30	0.89	14.24	−27.07	28.85	.950
Sex: male	3.65	8.58	−13.20	20.50	.671
BMI (kg/m^2^)					
Underweight (BMI < 18.5)	−46.58	37.20	−119.63	26.47	.211
Overweight (25 ≤ BMI < 30)	7.54	9.68	−11.47	26.55	.436
Obese (BMI ≥ 30)	26.34	13.18	0.45	52.22	**.046**
Ever smoking	11.34	9.06	−6.45	29.12	.211
Currently smoking	9.36	14.53	−19.17	37.89	.520
Morbidities					
Hypertension	5.55	10.66	−15.40	26.49	.603
Diabetes mellitus	54.85	24.17	7.39	102.31	**.024**
Hyperlipidemia	2.47	12.48	−22.05	26.98	.843

Variables linked with the elevation of BEC were examined through multivariable linear regression analyses including all the variables presented in the table. Data are presented as the unstandardized *B* coefficient, SE of *B*, and 95% CI. The age group 30-60 y was used as the reference for age variables, and the normal weight range (20 ≤ BMI < 25) served as the reference for BMI-related variables. Boldfaced entries signify *P* < .05 which was considered significant.

Subsequently, to discern factors linked to higher BEC values in the absence of established morbidities known to increase BEC, such as atopy, clinical allergy, asthma, and COPD, we conducted a separate analysis within this subset, in which diabetes and obesity were identified as significant predictors of BEC when adjusted for age and BMI as categorical variables (Table IV). When age and BMI were treated as continuous variables, BMI was significant and diabetes and ever smoking showed a trend toward significance (Table E3).

Finally, we conducted multivariable linear regression analyses separately in patients with asthma, COPD, and clinical allergy. Asthma remained significant in patients with clinical allergy, and clinical allergy showed a strong trend toward significance in patients with asthma. In addition, we found that age significantly influences BEC in the asthma group, unlike in the other groups (see Table E4 in this article’s Online Repository at www.jaci-global.org).

### Binary logistic regression model for factors associated with elevated BEC values

**Fig 3:**
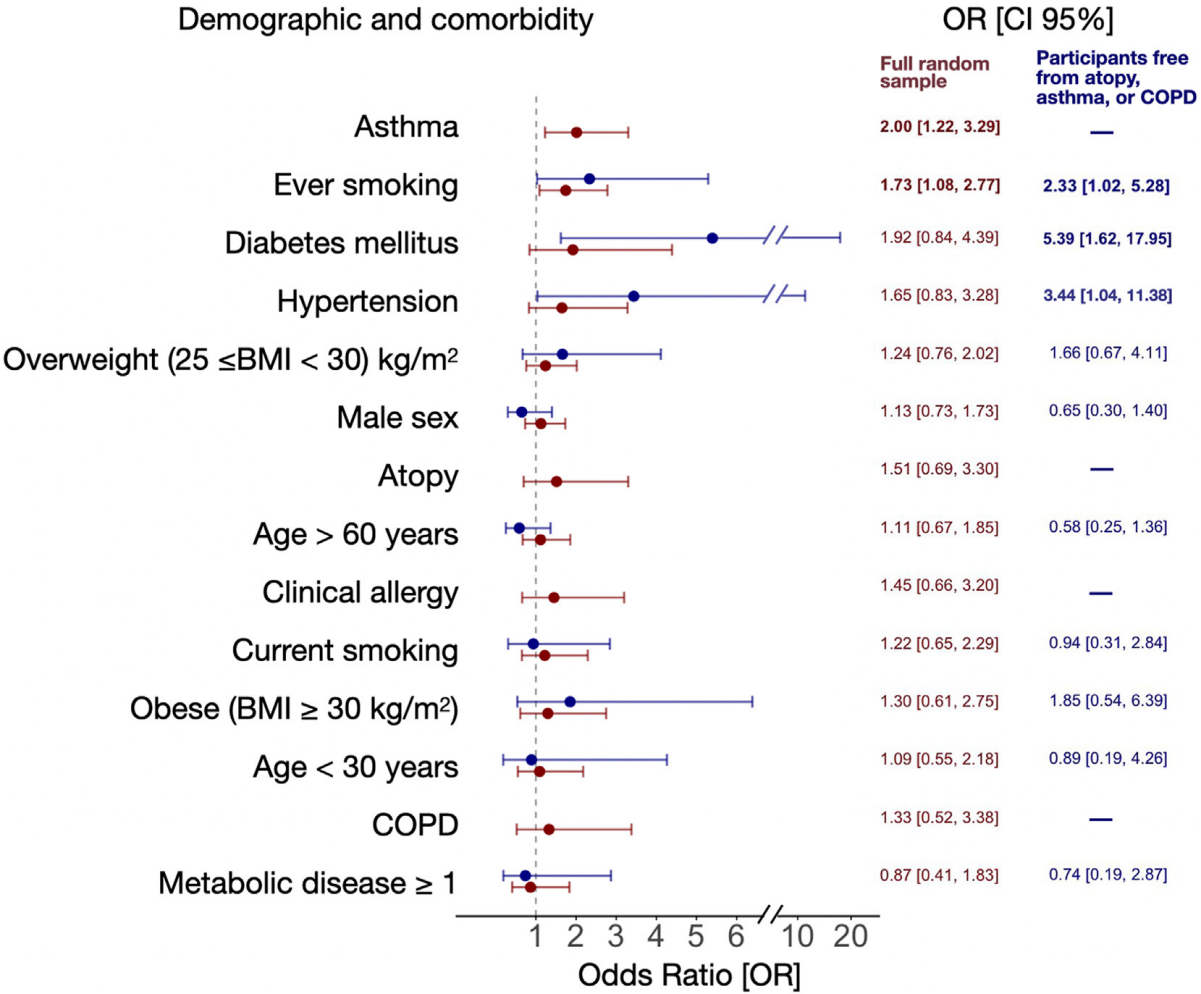
Adjusted binary logistic regression analyses (OR and 95% CI) for BEC greater than 400 cells/μL in the full population-representative random sample (n = 1145; *red*) and in subjects without clinical allergy, atopy, asthma, and COPD (n = 628; *blue*). Bold font indicates significant OR (*P* < .05) associations. The age group of 30 to 60 years was used as the reference for age variables, whereas the normal weight range (20 ≤ BMI < 25) served as the reference for BMI-related variables. *OR*, Odds ratio.

To evaluate factors associated with BEC higher than the upper limit of normal (>400 cells/μL), we conducted adjusted binary logistic regression analyses similar to the aforementioned linear regressions. In the whole sample, asthma and ever smoking were significantly associated with high BEC (Fig 3). Diabetes, hypertension, and ever smoking were significantly associated with high BEC in participants without atopy, asthma, or COPD (Fig 3).

## Discussion

In our population-representative random sample, the upper limit of BEC ranged from 400 to 500 cells/μL depending on age. In individuals without asthma, COPD, or clinical allergy, the upper limit of BEC increased from 300 to 400 cells/μL after the age of 40 years. Sex, smoking, atopy, clinical allergy, obesity, asthma, COPD, diabetes, and hypertension were significantly associated with higher BEC levels. However, only asthma and clinical allergy in the full sample, and obesity and diabetes in healthy individuals, remained significant determinants of BEC. Asthma and ever smoking were associated with high BEC in all subjects, whereas diabetes, hypertension, and ever smoking were associated with high BEC in healthy participants. These findings clarify the highly needed typical BEC range in the general population, a crucial biomarker for diagnosing, treating, and managing asthma and COPD.^1^^,^^2^

Several studies have attempted to establish reference values for BEC in their populations.^20^^,^^30^, ^31^, ^32^, ^33^, ^34^ The upper limit of normal BEC ranged from 550 to 630 cells/μL in a French population,^31^ from 500 to 600 cells/μL in a Moroccan population,^33^ and from 450 to 590 cells/μL in a Turkish population,^30^ depending on sex and age. In healthy individuals, the upper limit ranged from 500 to 800 cells/μL in a Thai population^32^ and from 490 to 640 cells/μL in a Kenyan population,^34^ depending on sex. Even though the upper limit of normal BEC in our study similarly varied by age with relatively lower range, it is important to note that most of these studies do not represent the general population accurately, and their outcomes could be affected. They analyzed data from either selected registries or small sample sizes instead of the large randomly selected sample from the general population. In addition, the studies using healthy populations have applied impractical strict criteria in defining their healthy population, such as excluding obese individuals, smokers, alcohol consumers, or participants with any comorbidities. In particular, our findings align more closely with those of Hartl et al,^20^ who reported a 95th percentile BEC range of 360 to 450 cells/μL in the general population and 290 to 350 cells/μL in healthy individuals, using a large sample size.

The reference intervals for BEC used in diverse contexts exhibit variability, encompassing upper limits ranging from the 75th to the 90th, the 95th, and as high as the 97.5th percentile.^20^^,^^30^, ^31^, ^32^, ^33^, ^34^, ^35^, ^36^ Hartl et al^20^ designated the 75th percentile as a threshold denoting “high blood eosinophils,” using it in their forced-entry binary multivariable logistic regression analysis. However, our analysis reveals that the sensitivity of the 75th percentile to alterations is somewhat diminished, contingent upon factors such as age, atopy, clinical allergy, and smoking. Conversely, the 95th percentile exhibited greater sensitivity to changes in these factors. In addition, using the 75th percentile to label something as “high” implies that 25% of healthy adults are abnormal, which is not reasonable. Even those above the 95th percentile do not necessarily have a disease. However, their significant deviation from the norm suggests a higher likelihood of clinically relevant abnormality. Consequently, we used the 95th percentile as the basis for our adjusted binary logistic regression analysis.

The median of BEC as reported in both healthy and general population studies exhibits a range of 100 to 200 cells/μL,^8^^,^^13^^,^^14^^,^^23^^,^^37^^,^^38^ with a proclivity to approximate 200 cells/μL in investigations with large sample sizes.^8^^,^^21^^,^^23^ Consistent with existing literature, our study observed a distribution of random sample BEC medians ranging from 100 cells/μL in the younger than 30 years age group to 200 cells/μL in the older age strata. Moreover, in concordance with asthma population studies, the median BEC has been reported to fluctuate between 157 and 298 cells/μL,^6^^,^^7^^,^^15^^,^^16^^,^^21^^,^^37^^,^^39^^,^^40^ demonstrating a tendency to approximate 200 cells/μL in studies involving a sizable participant pool.^6^^,^^7^^,^^15^^,^^21^ In our study, the median BEC varied from 180 cells/μL among asthmatic participants younger than 30 years to 200 cells/μL in the remaining age strata. Although the absolute and average BEC values are parallel to those in other cohorts, our study uniquely helps determine the abnormality of a person’s measured value by providing reference values from a representative sample of the general population.

In our study, when comparing BEC levels between groups, we found that male sex, smoking (both current and past), atopy, clinical allergy, obesity, asthma, COPD, diabetes, and hypertension were significantly associated with higher BEC levels. Previous studies have reported similar findings regarding male sex,^20^^,^^21^^,^^23^^,^^30^^,^^36^^,^^37^ current smoking,^20^^,^^36^^,^^37^ ever smoking,^36^ metabolic disorders,^20^^,^^23^^,^^30^ and allergy parameters.^20^^,^^23^^,^^36^ Caspard et al^37^ found no significant difference between patients with COPD and those without COPD despite the fact that they did find a significant association between higher BEC levels in current smokers in their non-COPD cohort. However, they used a wide self-reported definition of COPD, whereas we assessed patients with COPD on the basis of smoking history and clinical spirometry measurements.

Our study is the first to characterize the determinants of BEC levels using multivariable linear regression, showing that asthma and clinical allergy were significant predictors in the full random sample, whereas obesity and diabetes were significantly associated in healthy individuals. In addition, BMI was a significant predictor in both settings when treated as a continuous variable (Table E3). Giovannelli et al^23^ also identified allergic asthma, atopy, and BMI as predictors of BEC in their cohort, although BMI was significant only among nonsmokers. However, there was no analysis for predictors in the healthy subgroup in their study. In addition, no previous study has explored the impact of clinical allergy on BEC within the general population; most studies have evaluated the influence of atopy. This is of particular significance given that clinical allergy retains its status as a substantive predictor of BEC even after adjustments for several confounders including atopy in our multivariable linear regression analysis. Moreover, clinical allergy, rather than atopy alone, is used to define type 2 inflammation in asthma guidelines.^2^

The WSAS cohort presents several notable strengths. First, the study encompasses a sizable sample, systematically selected without any exclusion criteria, rendering it representative of the adult population in Western Sweden. Second, our investigation marks the first exploration of the impact of clinical allergy, in conjunction with atopy, among the participants. Challenges often arise in diagnosing factors such as clinical allergy and asthma, with many studies relying on self-reported disease. In contrast, our study used a meticulous classification process, involving thorough clinical examinations overseen by pulmonary and allergy specialists. This approach enhances the reliability of our findings, which reveal that although asthma and clinical allergy are predictors of BEC in the general population, only asthma and a history of smoking are linked to BEC levels higher than the upper normal limit. The fact that allergic parameters do not pose a risk for abnormal BEC values underscores the potential utility of our established upper limit of normal for interpreting BEC values in asthma clinics. Nevertheless, it is crucial to note the limitation of the present study to adults, acknowledging that the predicted BEC values might be substantially different among children and adolescents. In addition, our study does not account for less common infectious or chronic diseases that may be linked to elevated BEC, and nor does it consider ethnicity.

The upper limit of normal BEC in participants without asthma, COPD, and clinical allergy ranged between 300 and 400 cells/μL depending on age. The Global INitiative for Asthma recommends the use of BEC to identify asthmatic patients with type 2 inflammation using cutoff points such as 150 and 300 cells/μL.^2^ Similarly, the Global Strategy for the Diagnosis, Management and Prevention of Chronic Obstructive Lung Disease recommends the use of 300 cells/μL as a threshold of BEC to guide therapy with ICSs in patients with COPD.^1^ It is important to note that these cutoff points fall within the normal BEC range for healthy adults in our cohort, indicating that although they are useful for predicting treatment response, they are not necessarily considered “high.” However, the observation that only asthma and smoking, but not clinical allergy, are associated with abnormal BEC values highlights the potential for establishing treatment response indicators for asthma and COPD that align with reference values for healthy individuals.

Depending on age, the 95th percentile in BEC ranged between 400 and 500 cells/μL in a random sample of the general adult population and between 300 and 400 cells/μL in individuals free from asthma, COPD, and clinical allergy. In addition, asthma and smoking, although not allergic parameters, were identified as risk factors for having BEC levels higher than the upper limit of normal in the full sample. In subjects without asthma, clinical allergy, or COPD, smoking, diabetes, and hypertension were recognized as risk factors for elevated BEC. Consequently, these factors merit careful consideration during the clinical interpretation of BEC.

**Clinical implications**

The upper limit of normal BEC in participants free from asthma, COPD, and clinical allergy ranged between 300 and 400 cells/μL depending on age. These results facilitate the interpretation of BEC in clinical practice.

## Disclosure statement

The study was supported by the VBG Group Herman Krefting Foundation for Asthma and Allergy Research (Trollhättan, Sweden), the 10.13039/501100004359Swedish Research Council (Stockholm, Sweden), the 10.13039/501100003793Swedish Heart-Lung Foundation (Stockholm, Sweden), the Swedish Asthma and Allergy Foundation (Stockholm, Sweden), the 10.13039/501100006706Tampere Tuberculosis Foundation (Tampere, Finland), and the ALF agreement (grant from the Swedish state under the agreement between the Swedish government and the county councils [Sweden]).

Disclosure of potential conflict of interest: L. Lehtimäki reports personal fees from ALK, AstraZeneca, Berlin Chemie, Boehringer Ingelheim, Chiesi, GlaxoSmithKline, Novartis, Orion Pharma, and Sanofi, outside the submitted work. S. S. Ö. Ermis reports conference-attendance–related costs from Thermo Fisher Scientific, outside the submitted work. T. Pullerits reports fees for lectures and/or consulting from AstraZeneca, Chiesi, GlaxoSmithKline, Novartis, and Sanofi, outside the submitted work. H. Backman reports personal fees for lectures from AstraZeneca, Boehringer Ingelheim, and GlaxoSmithKline, outside the submitted work. B. I. Nwaru reports personal fees for lectures and consulting from DBV Technologies and AstraZeneca, outside the submitted work. H. Kankaanranta reports fees for lectures and/or consulting from AstraZeneca, Boehringer Ingelheim, Chiesi, Covis Pharma, GlaxoSmithKline, MedScape, MSD, Novartis, Orion Pharma, and Sanofi, outside the submitted work. The rest of the authors declare that they have no relevant conflicts of interest.
